# The effect of respiration buffer composition on mitochondrial metabolism and function

**DOI:** 10.1371/journal.pone.0187523

**Published:** 2017-11-01

**Authors:** Lucas C. Wollenman, Matthew R. Vander Ploeg, Mackinzie L. Miller, Yizhu Zhang, Jason N. Bazil

**Affiliations:** 1 Department of Physiology, Michigan State University, East Lansing, MI, United States of America; 2 Vanderbilt University School of Medicine, Nashville, TN, United States of America; 3 Biomedical Laboratory Diagnostics, Michigan State University, East Lansing, MI, United States of America; 4 Nephrology and Hypertension, Henry Ford Hospital, Detroit, MI, United States of America; University of Mississippi, UNITED STATES

## Abstract

Functional studies on isolated mitochondria critically rely on the right choice of respiration buffer. Differences in buffer composition can lead to dramatically different respiration rates leading to difficulties in comparing prior studies. The ideal buffer facilities high ADP-stimulated respiratory rates and minimizes substrate transport effects so that the ability to distinguish between various treatments and conditions is maximal. In this study, we analyzed a variety of respiration buffers and substrate combinations to determine the optimal conditions to support mitochondrial function through ADP-stimulated respiration and uncoupled respiration using FCCP. The buffers consisted of a standard KCl based buffer (B1) and three modified buffers with chloride replaced by the K-lactobionate, sucrose, and the antioxidant taurine (B2) or K-gluconate (B3). The fourth buffer (B4) was identical to B2 except that K-lactobionate was replaced with K-gluconate. The substrate combinations consisted of metabolites that utilize different pathways of mitochondrial metabolism. To test mitochondrial function, we used isolated cardiac guinea pig mitochondria and measured oxygen consumption for three respiratory states using an Oroboros Oxygraph-2k. These states were the leak state (energized mitochondria in the absence of adenylates), ADP-stimulated state (energized mitochondria in the presence of saturating ADP concentrations), and uncoupled state (energized mitochondria in the presence of FCCP). On average across all substrate combinations, buffers B2, B3, and B4 had an increase of 16%, 26%, and 35% for the leak state, ADP-simulated state, and uncoupled state, respectively, relative to rates using B1. The common feature distinguishing these buffers from B1 is the notable lack of high chloride concentrations. Based on the respiratory rate metrics obtained with the substrate combinations, we conclude that the adenine nucleotide translocase, the dicarboxylate carrier, and the alpha-ketoglutarate exchanger are partially inhibited by chloride. Therefore, when the goal is to maximize ADP-stimulated respiration, buffers containing K-lactobionate or K-gluconate are superior choices compared to the standard KCl-based buffers.

## Introduction

Mitochondrial respiration involves the consumption of oxygen in a process that converts the chemical energy stored in carbon substrates into a chemical energy that is useable by the cell called the ATP hydrolysis potential or ATP phosphorylation potential [1]. This process involves many steps and enzymes located inside the mitochondrial matrix, on the inner membrane, in the intermembrane space, and even on the outer membrane. When mitochondria oxidize substrates, they utilize various catabolic pathways that converge on oxidative phosphorylation to produce ATP. This ability to oxidize a variety of substrates allows the mitochondria to provide the cell with ATP under a wide range of conditions. Additionally, some catabolic pathways provide more efficient forms of chemical energy transformation than other pathways, and the environmental factors that support these more efficient pathways are under active study [2–7]. Therefore, the mitochondrial environment is an important factor whenever investigating changes in mitochondrial respiration due to drug toxicity or disease conditions as effector molecules within the environment can increase or decrease respiration and ATP production rates to varying degrees. Recapitulating the proper mitochondrial environment *in vitro* is essential to extrapolate to the *in vivo* condition [8, 9], as a near-physiological setting is desired for isolated mitochondrial investigations. In addition, comparative mitochondrial assays require conditions that maximize the differences between treatments and conditions. This is achieved by using a respiration buffer that yields better-coupled and functional mitochondria and doesn’t inhibit mitochondrial processes that could interfere with the interpretation of the data.

In this study, we tested mitochondrial function in four types of respiratory buffers. The first buffer (B1) is a common KCl-based buffer used in many mitochondrial respiratory studies [10–14], which presumably better represents the ionic environment of the cytosol relative to sucrose-based respiration buffers. The second buffer (B2) is a hybrid buffer mainly consisting of sucrose, K-lactobionate, and taurine, which consists of a mixture of ionic, non-ionic, stabilizing, and antioxidant constituents. This buffer has been used in other mitochondrial studies [15–17], but we are not aware of a direct mitochondrial functional analysis comparing the hybrid buffer with KCl-based buffers. Lactobionate is a disaccharide that possesses cytoprotective properties [18] and like other impermeant anions, prevents cell and mitochondrial swelling. It also seems to have beneficial effects on mitochondrial function and integrity for which the mechanisms are not well known [19]. In addition, lactobionate acts as a calcium-chelating agent, binding calcium with a high affinity [18, 20–22]. Taurine acts as an antioxidant, scavenging free radical species generated by mitochondria, and is thought to be involved with membrane stabilization, osmoregulation, and ion channel regulation [23]. The third buffer (B3) consists of similar composition to that of buffer B1, but the KCl is replaced with K-gluconate. Gluconate is a carboxylic acid with metal chelating properties and has been used in some mitochondrial respiration buffers [24, 25]. The fourth buffer (B4) is similar to B2 but the K-lactobionate is replaced with K-gluconate. A notable difference that distinguishes buffers B2, B3, and B4 from B1 is the amount of chloride they contain. Buffer B1 contains unphysiologically high levels of chloride concentrations that can detrimentally alter some mitochondrial processes [26–30]. Buffer B3 avoids this problem by replacing nearly all the chloride with gluconate. In addition, buffers B2 and B4 also contain lower chloride but are lower in ionic strength, which may also affect mitochondrial respiration [31].

We found that the buffers containing low chloride concentrations led to higher ADP-stimulated respiratory rates across a wide variety of substrates. This effect is likely due to higher activities of mitochondrial solute carriers including the dicarboxylic acid transporter, adenine nucleotide translocase, and possibly the alpha-ketoglutarate/malate exchanger. In addition, higher respiratory control ratios (the ratio of maximally ADP-stimulated respiration over the leak or resting state condition) were observed for most of the substrates tested. This is indicative of better-coupled mitochondria and presumably better functioning mitochondria. Thus, we recommended using these types buffer as the preferred choice for respiration studies, especially when maximizing respiration and ATP production is preferred.

## Methods

### Mitochondria isolation and protein quantification

The work presented herein conformed to the National Institutes of Health’s Guild for the Care and Use of Laboratory Animals and was approved by Michigan State University’s Institutional Animal Care and Use Committee. Hartley guinea pigs (4–6 weeks) were anesthetized with 5% isoflurane. When the animal was under a deep plane of anesthesia and unresponsive to noxious stimuli, it was decapitated and the heart was retrogradely perfused in-chest with ice-cold cardioplegia solution until mechanically silent and no blood remained in the coronary arteries and cardiac veins. The cardioplegia solution consisted of 25 mM KCl, 100 mM NaCl, 10 mM dextrose, 25 mM MOPS, and 1 mM EGTA at pH 7.15 and sterile filtered (0.22 μm). After flushed with cardioplegia, the hearts were excised and briefly washed with ice-cold isolation buffer to remove blood from the ventricles. The isolation buffer consisted of 200 mM mannitol, 50 mM sucrose, 5 mM dibasic potassium phosphate, 5 mM MOPS, 1 mM EGTA, and 0.1% (w/v) BSA at pH 7.15. The thymus, atria, great vessels, and fat were excised and both ventricles were minced in ice-cold isolation buffer with scissors until approximately 1 mm^3^ sized pieces remained. Then the heart pieces were transferred to a 50 mL tube with 0.5 U/mL of protease (Bacillus licheniformis) in 25 mL isolation buffer. The tissue was homogenized at 18,000 rpm for 20 sec using an Omni handheld homogenizer with a 110 mm probe. After homogenization, the sample was spun at 8,000 x g in a fixed angle, tabletop centrifuge for 10 min. The resulting pellet was washed with isolation buffer and resuspended in 25 mL isolation buffer by vortexing. Then the sample was spun at 800 x g, and the supernatant was collected in a fresh 50 mL tube. A final 8,000 x g spin was done to pellet the purified mitochondria. All centrifugations were carried out at 4°C. Afterwards, the pelleted mitochondria were resuspended in 100–150 μl isolation buffer, and the protein was quantified using the BCA assay using bovine serum albumin standards. Absorbance was quantified using an Olis DM245 spectrofluorimeter with a dual-beam absorbance module (Olis, Inc., Bogart, GA). The mitochondrial suspension was typically diluted to a 40 mg/mL stock concentration. For the preparation of liver mitochondria, the liver was excised from the animal after the thoracotomy and washed of blood with ice-cold isolation buffer. The tissue was then minced with scissors, homogenized, and processes as done for the cardiac mitochondria.

### Respiration buffer composition

We tested the effect of four buffers on mitochondrial metabolism. The first buffer (B1) is an ionic-based buffer consisting of 130 mM potassium chloride, 5 mM dibasic potassium phosphate, 1 mM magnesium chloride, 20 mM MOPS, 1 mM EGTA, 0.1% (w/v) BSA at pH 7.1 at 37°C. The second buffer (B2) is a hybrid buffer consisting of 110 mM sucrose, 60 mM potassium lactobionate, 20 mM taurine, 10 mM monobasic potassium phosphate, 3 mM magnesium chloride, 20 mM HEPES, 1 mM EGTA, and 0.1% (w/v) BSA at pH 7.1 at 37°C. This buffer is from the MiR05 recipe [19]. The third buffer (B3) consists of 130 mM potassium gluconate, 5 mM dibasic potassium phosphate, 1 mM magnesium chloride, 20 mM MOPS, 1 mM EGTA, and 0.1% (w/v) BSA at pH 7.1 at 37°C. The fourth buffer (B4) consists of 110 mM sucrose, 60 mM potassium gluconate, 20 mM taurine, 10 mM monobasic potassium phosphate, 3 mM magnesium chloride, 20 mM HEPES, and 0.1% (w/v) BSA at pH 7.1 at 37°C. To test the effect of taurine on mitochondrial respiration, two additional buffers were used. The first was identical to the B1 buffer; however, this buffer contained taurine. To keep the osmolarity similar, the potassium chloride concentration was lowered. The buffer composition was 110 mM potassium chloride, 20 mM taurine, 5 mM dibasic potassium phosphate, 1 mM magnesium chloride, 20 mM MOPS, 1 mM EGTA, and 0.1% (w/v) BSA at pH 7.1 at 37°C. The second buffer used to test the effect of taurine was identical to B2 but without taurine. For this buffer, the osmolarity was kept the same by supplementing the modified B2 buffer with extra sucrose. This buffer consisted of 130 mM sucrose, 60 mM K-lactobionate, 10 mM monobasic potassium phosphate 3 mM magnesium chloride, 20 mM HEPES, 1 mM EGTA, and 0.1% (w/v) BSA at pH 7.1 at 37°C. For all buffers, pH was adjusted with KOH. To test the effect of calcium on respiration, buffers B1 and B2 were made without EGTA. However, low levels of EGTA (~2.5 μM) are carried over from the isolation buffer when mitochondria are added to the respiratory chambers. All reagents were from Sigma unless otherwise noted.

### Respirometry and quality control

All mitochondrial respiration data were collected at 37°C using an Oxygraph 2k (Oroboros Instruments GmbH, Innsbruck, Austria). A five-point averaging window was used to calculate the rate of oxygen consumption from the oxygen concentration data. The quality of mitochondria was determined from the respiratory control ratio (RCR) defined as the rate of oxygen consumption at saturating ADP and substrate concentrations (ADP-stimulated rate) divided by the rate of oxygen consumption at saturating substrate concentration in the absence of nucleotides (leak state). The leak state rate was quantified by averaging the respiration rate for a 30-second window just prior to ADP addition. The ADP-stimulated rate was quantified by averaging over an approximately 30-second window covering the peak respiration dynamic. Mitochondria were loaded in the oxygraph chambers at a concentration of 0.1 mg/mL in respiration buffer and energized with 5 mM sodium pyruvate and 1 mM neutralized malic acid. For the RCR measurements, buffer B1 was used, and the values ranged from 19–23. The free calcium concentration in the presence of 1 mM EGTA was expected to be approximately less than 10 nM. Values presented are uncorrected oxygen consumption rates. We calculated that the average oxygen consumption rate due to the electrodes in each chamber was 0.52 +/- 4.20 pmol O_2_/s/mL. Electrode calibration was performed daily before each use.

### Calcium contamination quantification

Free calcium concentrations in the respiration buffers were measured using a perfectION^TM^ calcium selective electrode (Mettler Toledo, Columbus, OH) using the low-level calcium calibration and measurement method.

### Respiration protocol

Mitochondrial respiration was measured in the presence of a variety of substrates (Table 1) under the conditions outlined above. These combinations consist of all the common combinations including a few others to test different effects of buffer composition on mitochondrial metabolism. The mitochondrial protein concentration was 0.1 mg/ml for the cardiac mitochondria and 0.5 mg/ml for the liver mitochondria. Oxygen consumption was recorded in the leak state, ADP-stimulated state, and an FCCP-stimulated uncoupled state. For the ADP-stimulated, 500 μM of neutralized ADP was injected into the chambers. For uncoupled respiration measurements, FCCP was injected into the chamber in 0.5 μM increments until the maximum respiration rate was attained. The uncoupled respiration experiments were done separately from the ADP-stimulated respiration experiments.

**Table 1:** Substrate combinations.

Substrate Code	Substrates^a^	Concentrations
P/M	pyruvate / malate	5 mM / 1 mM
G/M	glutamate / malate	5 mM / 5 mM
αKG/M	alpha-ketoglutarate / malate	5 mM / 1 mM
αKG/G	alpha-ketoglutarate / glutamate	5 mM / 5 mM
PC/M	palmitoylcarnitine / malate	25 μM / 2 mM
P/M/S	pyruvate / malate / succinate	5 mM / 1 mM / 10 mM
S/Rot	succinate / rotenone	10 mM / 0.5 μM

^a^The substrate stock solutions were made from sodium pyruvate, L-malic acid, L-glutamic acid, disodium alpha-ketoglutarate, palmitoyl-DL-carnitine chloride, and disodium succinate.

### Statistics

Results are expressed as mean +/- standard deviation. Data were checked and confirmed for normality using the Shapiro-Wilk test. Statistical significance was tested using an unbalanced, one-way ANOVA followed by a post-hoc Tukey’s range test for the respiration and RCR comparisons for buffers B1, B2, B3, and B4. An unbalanced, two-way ANOVA followed by a post-hoc Tukey’s range test was used to test the significance for the studies comparing the effectiveness of taurine. The statistical significance for the effect of buffers B1 and B2 on liver mitochondrial respiration was done using a t-test assuming equal variance. The *n* values ranged from 3 to 10 for each substrate combination and buffer. At least 3 biological replicates were used for each condition. The error bars for FCCP-stimulated/ADP-stimulated, calcium-effect, and percent ADP- and FCCP-stimulated rate data were computed using standard error propagation techniques [32].

## Results

**Fig 1:**
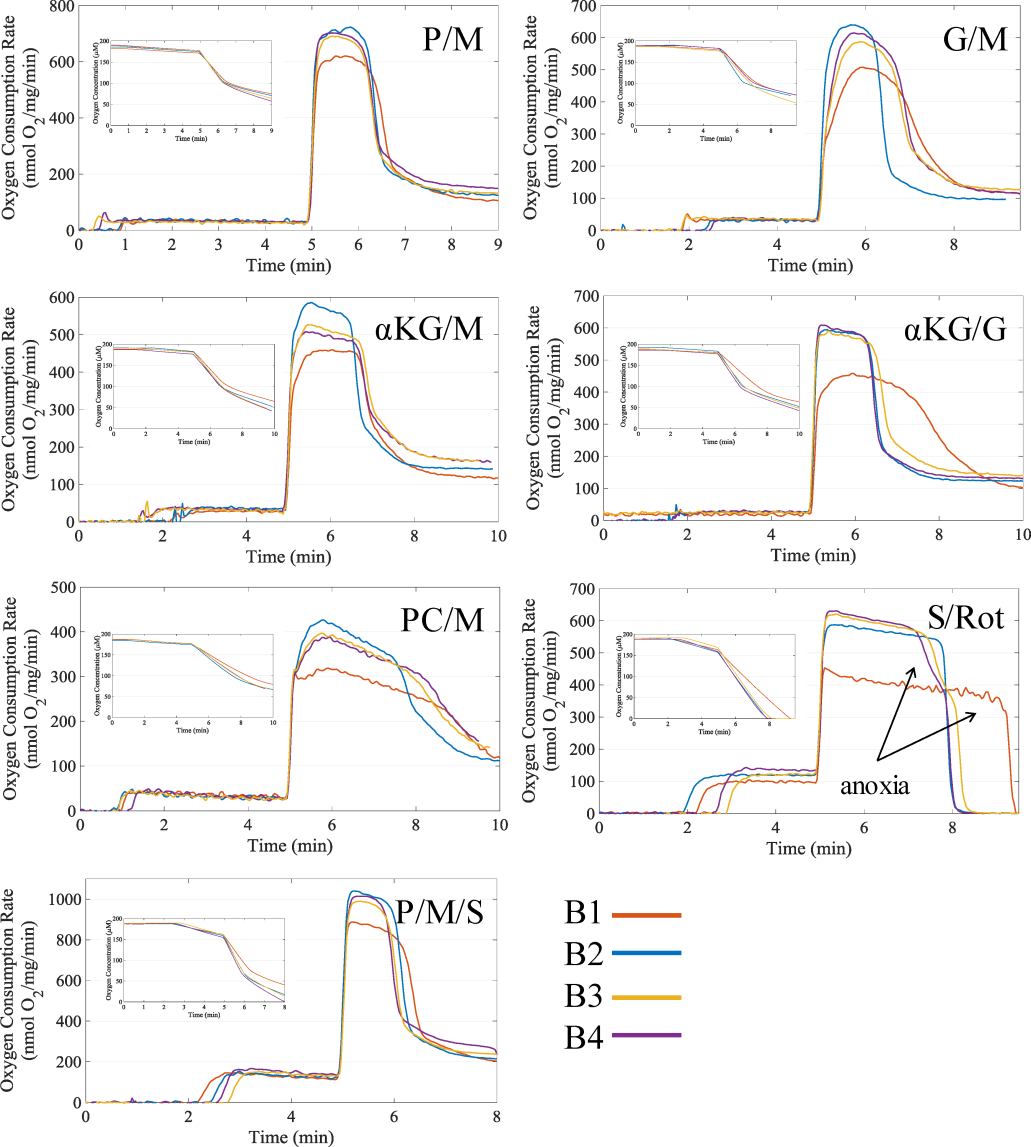
Representative respiration dynamics for each tested buffer and substrate combination. When leak state respiration stabilized, 500 μM ADP was added to initiate oxidative phosphorylation. The elevated respiration rates after ADP phosphorylation are due to variable contamination of ATPases. Respiration dynamics for P/M, G/M, αKG/M, αKG/G, PC/M, P/M/S, and S/Rot supported respiration are shown. See Table 1 for the final concentration used for each substrate. The respiration dynamics are aligned to the time of ADP addition at five minutes. The insets give the corresponding oxygen concentration data.

The oxygen consumption dynamic profiles as shown in Fig 1 were similar for all buffers for a given substrate combination. There were no major apparent differences other than higher ADP-stimulated respiration rates in the buffers with lower chloride concentrations (B2, B3, and B4). The leak state respiration rate was similar between buffers with very few significant differences except for succinate-dependent respiration rates. For non-succinate substrate combination, the rate was between 20 to 35 nmol O_2_/mg/min, and for succinate containing substrate combinations, the rate was higher between 100 to 150 nmol O_2_/mg/min. In general, buffer B1 led to the lowest respiration rates for any substrate combination. Across all substrate combinations, the combination that yielded the highest ADP-stimulated respiration was P/M/S. (For the substrate shorthand notation list, see Table 1) This was expected considering both the NADH and UQH_2_ electron pathways in the TCA cycle are more fully engaged. Without succinate supplementation, succinate dehydrogenase is substrate limited. This is because the entire TCA cycle is not completely functional as TCA cycle intermediates such as citrate and alpha-ketoglutarate are exchanged with malate by the tricarboxylate carrier and alpha-ketoglutarate/malate exchanger, respectively [33]. The P/M combination lead to the next highest ADP-stimulated respiration and was closely followed by the αKG/G, G/M, and S/Rot combinations. Relative to the other substrate combination profiles, the G/M, αKG/M, and αKG/G (for only B1) combination respiratory profiles showed a substrate- and buffer-dependent delay ranging from 20 to 50 sec until the maximum ADP-stimulatory rate was achieved. This delay was not present when low concentrations of free calcium was present (see S1 Fig). The last two substrate combinations in order of maximal ADP-stimulated respiration rates were αKG/M and PC/M.

**Fig 2:**
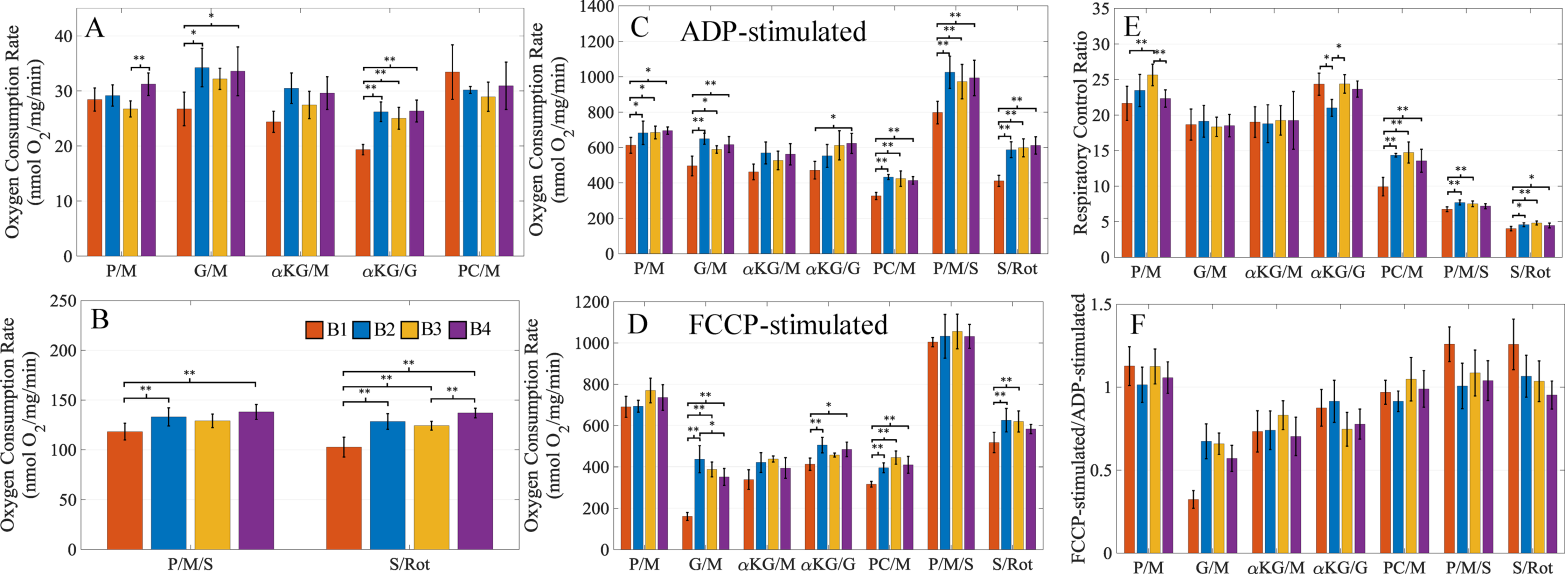
Comparison of the buffers B1, B2, B3, and B4. (A) The leak state respiration rates for non-succinate substrate combinations. (B) The leak state respiration rates for succinate substrate combinations. (C) The ADP-stimulated respiration rates for all substrate combinations. (D) The FCCP-stimulated uncoupled respiration rates for all substrate combinations. (E) The respiratory control ratios (ADP-stimulated rate/leak rate) for all substrate combinations. (F) The electron transport system capacity as measured by the maximally FCCP-stimulated rate over the ADP-stimulated rate. Error bars for (F) are propagated standard deviations. Leak state respiration rates were averaged over a 30-second window just prior to ADP addition. ADP-stimulated and FCCP-uncoupled respiration rates were averaged over a 30-second window covering the peak of each respiration dynamic. Data presented as mean +/- standard deviation (*n* values ranged from 3–8 for B1, 3–8 for B2, 3–9 for B3, and 3–8 for B4). A * indicates a significance level p<0.05, and a ** indicates significance level of p<0.01.

A closer examination of the respiratory rates highlights the differences between the buffers. Fig 2 shows that the buffers had very similar leak state respiratory rates with few exceptions (Fig 2A and 2B). The differences in rates typically fall within a 20% margin relative to the rate obtained using B1. Among the substrate combinations, the rates for αKG/G and S/Rot showed the most significant differences between the buffers. However, most of these differences are when compared to buffer B1. A similar result appears when comparing the ADP-stimulated respiration rates across the substrate combinations for each buffer. These rates were generally higher across all substrate combinations for the buffers with lower chloride concentrations (Fig 2C). With these buffers, there were significantly higher ADP-stimulated respiration rates for five substrate combinations compared to the B1 buffer (P/M, G/M, PC/M, P/M/S, and S/Rot). These higher ADP-stimulated respiration rates resulted in higher respiratory control ratios (RCR) across most substrate combinations (Fig 2E). The αKG/G combination for buffer B2 RCR was lower due to a higher leak state respiratory rate. The cause of this is unknown. The FCCP uncoupled respiration rates for certain substrate combinations also showed higher rates for the buffers containing low chloride (Fig 2D). Specifically, the low chloride containing buffers led to higher FCCP-uncoupled rates for the G/M, αKG/G, PC/M, and S/Rot substrate combinations. We can rule out a direct inhibition of the electron transport system, as the FCCP-stimulated rates for the P/M and P/M/S substrate combinations are very similar. Based on our findings, chloride appears to inhibit mitochondrial function in at least two independent processes. The most likely targets of chloride inhibition are the adenine nucleotide translocase and substrate transporters involved with succinate, glutamate, and palmitoylcarnitine catabolism. Our data also suggest the transporter for alpha-ketoglutarate may also be inhibited, but the rates were not significantly different from buffer B1 for all conditions. These observations are also given in S2 Fig as a percent difference between buffers B2, B3, and B4 relative to B1.

Overall, the P/M, P/M/S, and S/Rot uncoupled respiration rates were typically higher than the maximal coupled respiration rates (Fig 2F). For PC/M, the maximal uncoupled rate was roughly equal to the maximal coupled rate. Surprisingly, the uncoupled rates were much lower than the coupled rates for the G/M, αKG/M, and αKG/G substrate combinations. The exact reason for this is unknown but may be related to FCCP interfering with substrate transport and/or inhibiting the electron transport system. During the FCCP-stimulated respiration experiments, 0.5 μM increments of FCCP were titrated into the chambers until the maximum respiration rate was reached. The FCCP concentration necessary to achieve maximum uncoupled respiration was substrate dependent and typically fell between 1 to 2 μM. We should note that the maximum concentration also depends on the concentration of mitochondria present in the chamber during the experiment. Many of the uncoupled experiments were performed in the adjacent respiratory chamber at the same time as the coupled experiments, and we noticed a consistently lower FCCP-stimulated rate compared to the ADP-stimulated rate for these substrate combinations. We believe FCCP poisoning of the respiratory chain is unlikely as higher rates of respiration were obtained with P/M, P/M/S, and S/Rot substrate combinations at higher FCCP dosages. With that said, a common solute carrier involved with the catabolism of the substrate combinations yielding lower uncoupled vs coupled respiration rates is the alpha-ketoglutarate/malate exchanger. We are unaware of any detailed studies looking at the effect of FCCP on this transporter’s activity; however, studies have shown FCCP can alter membrane properties [34] and the activity of this transporter is dependent on membrane fluidity [35]. Glutamate transport via the glutamate transporter or glutamate/aspartate carrier may also be effected by FCCP partitioning in the membrane.

**Fig 3:**
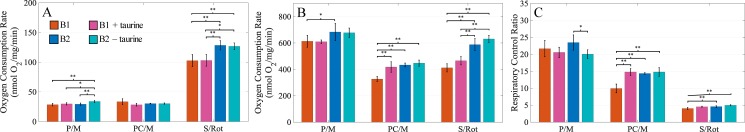
Comparing the effect of taurine on respiratory function. (A) The leak state respiration rates for P/M, PC/M, and S/Rot substrate combinations for the taurine containing and related taurine-free buffers are shown. (B) The ADP-stimulated respiration rates are shown. (C) The corresponding respiratory control ratios are shown. As with the data in Fig 2, the leak state respiration rates were averaged over a 30-second window just prior to ADP addition, and the ADP-stimulated respiration rates were averaged over a 30-second window covering the peak of each respiration dynamic. Data presented as mean +/- standard deviation (*n* values ranged from 3–4 for both buffers). A * indicates a significance level p<0.05, and a ** indicates significance level of p<0.01.

We then tested if the antioxidant taurine significantly contributed to the enhanced respiratory function of ADP-stimulated mitochondria. To do this, two additional buffers were tested. The first was identical to buffer B1 with the addition of taurine and the other was the B2 buffer without taurine. Fig 3 shows the comparisons of these buffers for the leak state, ADP-stimulated state, and RCR values for the substrate combinations P/M, PC/M, and S/Rot. For almost every buffer and substrate combination, there were no significant differences between the taurine and taurine-free buffers. The significant differences were all between the B1 class and B2 class buffers but for two exceptions. The first was a minor difference observed for the P/M dependent leak state respiratory rate for the B2 +/- taurine comparison, which lead to a difference in the RCR values. The second was seen with the B1 buffer for the PC/M substrate combination for both the ADP-stimulated respiratory rate and the corresponding RCR value. However, respiration on this substrate combination is quite sensitive to chloride (see Fig 2C and 2D), so the beneficial effect of the taurine supplementation could be because the chloride concentration was lower. Overall, this suggests that taurine does not play a significant role in the mitochondrial respiration differences observed between buffers.

**Fig 4:**
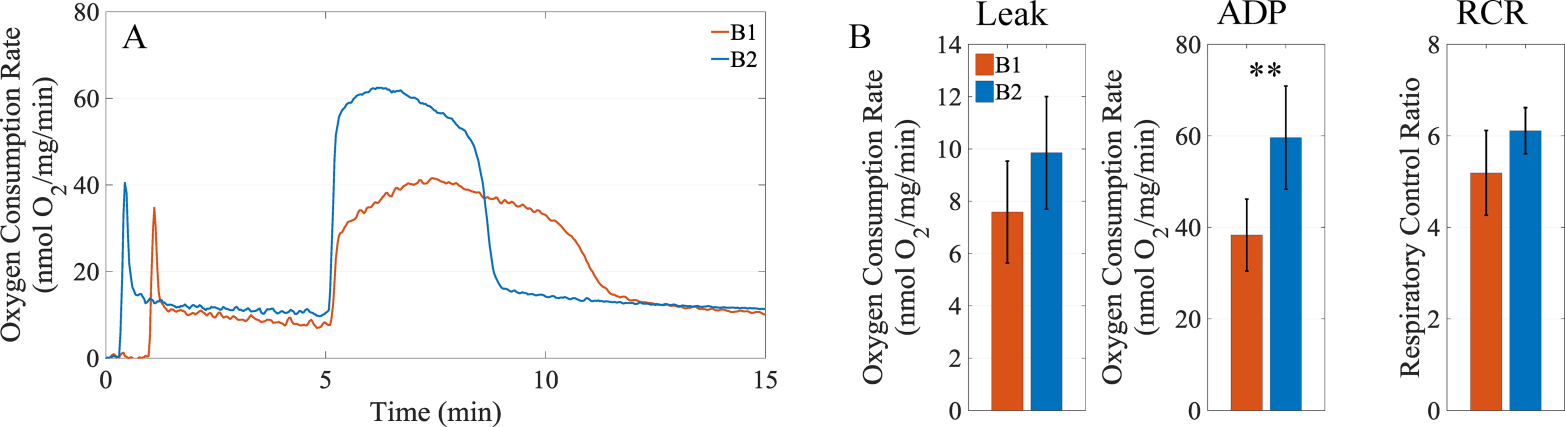
Comparing buffers B1 and B2 with liver mitochondria. Liver mitochondria were loaded in the oxygraph chambers at a concentration of 0.5 mg/ml. The substrate combination PM was used to test the effect of buffers B1 and B2 on respiration. (A) Representative dynamics for liver mitochondrial respiration in buffers B1 and B2. When leak state respiration stabilized, 500 μM ADP was added to simulate oxidative phosphorylation. (B) Comparison of leak state respiration rates, ADP-stimulated respiration rates, and RCRs. Leak state respiration rates were averaged over a 30-second window just prior to ADP addition. ADP-stimulated and FCCP-uncoupled respiration rates were averaged over a 30-second window covering the peak of each respiration dynamic. Data presented as mean +/- standard deviation; ** indicates significance level of p<0.01.

We next tested to see if the improved respiration performance of the low chloride containing buffers was specific to cardiac mitochondria by testing effects of buffers B1 and B2 on liver mitochondrial function. For this test, we used the P/M substrate combination. As shown in Fig 4A, the respiratory rates obtained with the buffer containing a low chloride concentration is higher than that of the buffer with a high chloride concentration. In general, the data reveal that buffer B2 leads to an increase of 30%, 56%, and 18% in the leak state respiration rate, ADP-stimulated rate, and computed the RCR, respectively, as shown in Fig 4B.

**Fig 5:**
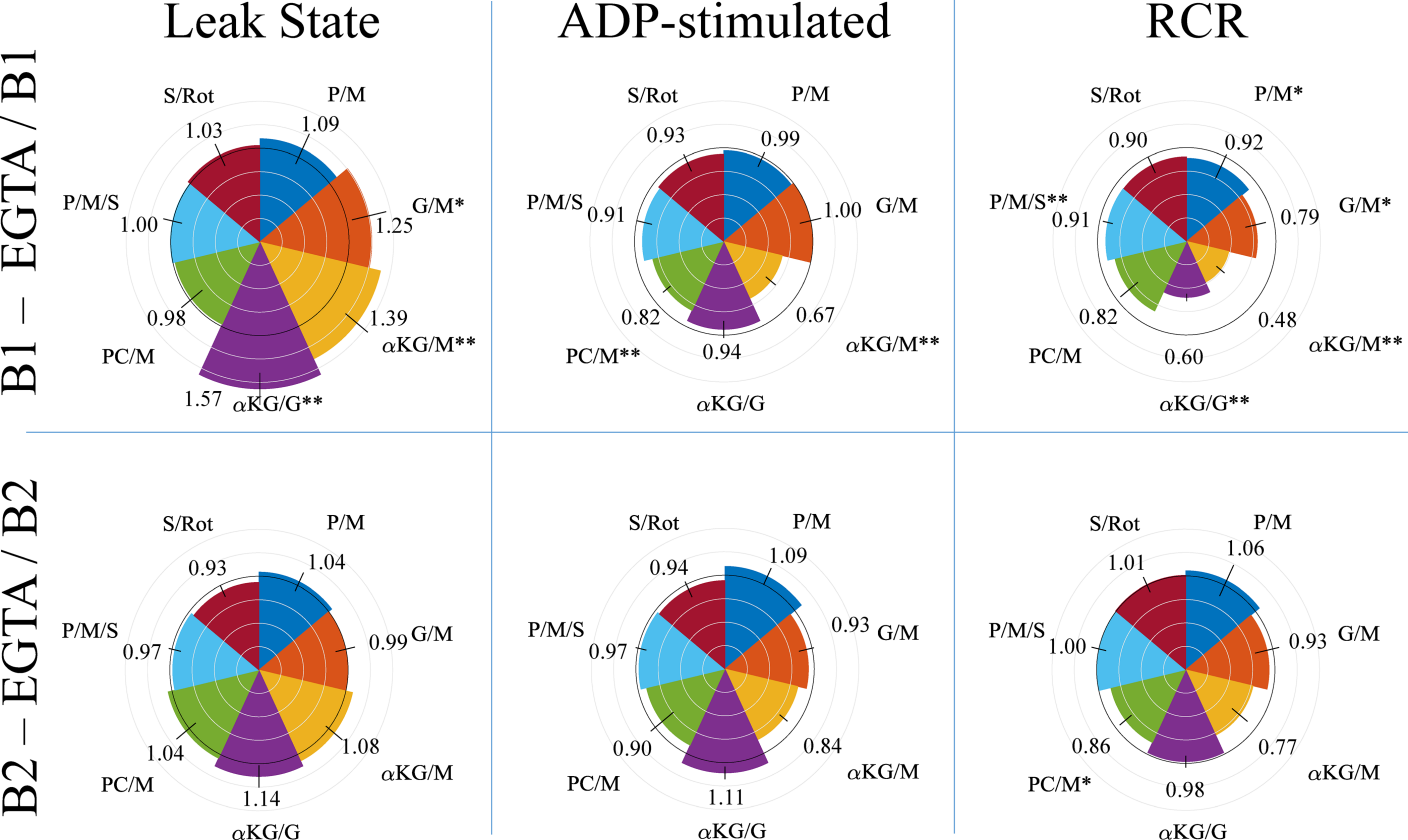
The effect of calcium on respiration with buffers B1 and B2. To measure the effect of calcium, the buffers were modified by removing EGTA from the constituents which resulted in a small amount of free calcium contamination. The first row shows the effect of calcium on leak state respiration, ADP-stimulated respiration, and the RCR for buffer B1. The second row shows the same mitochondrial variables for buffer B2. The black circle has a radius of one. Gray circles are radially spaced in 0.25 increments. Circular sectors that extend above or fall below the black inner circle for a given substrate arc indicate a positive or negative calcium effect, respectively. Data presented as the mean +/- standard deviation (*n* values ranged from 3–10 for B1 and 3–7 for B2). The mean data for each substrate are located near each circular sector, and the standard deviation is represented as a line segment perpendicular to the tangent for each substrate arc. Error bars are propagated standard deviations. A * indicates a significance level <0.05, and a ** indicates significance level of <0.01.

We also tested the effect of calcium-dependent respiration for buffers B1 and B2 by comparing the rates in the absence of EGTA with the rates observed in the presence of 1 mM EGTA as shown in Fig 5. Using a calcium sensitive electrode, we measured 5.7 +/- 0.51 μM (n = 5) of free calcium in B1 and 0.99 +/- 0.27 μM (n = 4) in B2 when EGTA was absent from either buffer. Calcium had varying effects on respiration rates in the respiratory trials, but its direct effect on metabolic pathways was greater than the effect sodium/calcium cycling. To deduce this, we note that leak state respiration rates for P/M, PC/M, P/M/S, and S/Rot were not significantly different between EGTA and non-EGTA buffers. As the P/M substrate combination contained 5 mM sodium, PC/M substrate combination contained 0 mM sodium, the S/Rot combination contained 20 mM sodium, and the P/M/S combination contained 25 mM sodium, if sodium/calcium cycling stimulated respiration in our experimental conditions, we would expect to see a difference between the EGTA and non-EGTA leak respiration rates. However, leak state respiration rates for B1 were significantly higher in the absence of EGTA with the well-known calcium dependent substrates G/M, αKG/M, and αKG/G. Therefore, the stimulatory effect of calcium on respiration from sodium/calcium cycling was small relative to the effect of calcium on the calcium-dependent metabolic pathways. While the substrate combination P/M showed a moderate calcium effect, the significance level is outside the 0.05 level. For buffer B2, the calcium effects were qualitatively similar but lower in magnitude. This is likely due to the lower free calcium concentration in this buffer. Two substrate combinations for the ADP-stimulated respiratory rates for B1 showed a significant calcium effect. With calcium present, respiration on PC/M and αKG/M showed a significant drop in the ADP-stimulated rate. The cause of these lower respiratory rates is unknown, but the effect was consistent across biological replicates. The calcium effects for the ADP-stimulated rates for buffer B2 were again qualitatively consistent compared with those seen with B1; however, the result generally show higher respiratory rates rather than lower. The calcium inhibitory effect seen with buffer B1 could have been alleviated by the lower calcium concentration for this buffer. The RCR values show a consistent negative effect of calcium for buffer B1 with all substrate combinations yielding lower RCR values in the absence of EGTA. As with the leak and ADP-stimulated respiratory rate results for buffer B2, the RCR comparisons show little to no calcium effect for most of the substrate combinations. The RCR values for the G/M, αKG/M, and PC/M substrate combinations decreased due to calcium, but the effect was only significant for the PC/M combination.

## Discussion

For the first time, to our knowledge, a comprehensive report on mitochondrial respiration rates for a variety of substrate combination and buffer formulations is given. In general, we found that buffers containing low chloride concentrations led to the highest rates of ADP-stimulated and FCCP-stimulated respiration across a wide variety of substrate combinations. In order to determine the key factor leading to the higher respiratory function, we systematically tested buffer formulations either consisting of or lacking likely constituents that contribute to the effect. In doing so, we found that the excessive amounts of chloride concentrations inhibited the adenine nucleotide translocase, in addition to, mitochondrial solute carriers involved with substrate transport. For comparison, reports of cytosolic chloride concentrations in heart muscle are variable but range from 5–30 mM [36–41]. A detailed discussion of our findings follows.

First, we note that our respiration results for the KCl-based buffer are corroborated by previous studies (see Table 2) on muscle mitochondria. For example, in mitochondria energized with P/M, our data for B1 are 28 +/- 2 nmol O_2_/mg/min, 618 +/- 43 nmol O_2_/mg/min, and 22 +/- 2 for leak state respiration, ADP-stimulated respiration, and the RCR, respectively. Previous studies with either P/M or P gave similar results for mitochondrial respiration rates [12, 14, 42, 43]. Prior G/M respiration rate values also coincide with our values of 27 +/- 3 nmol O_2_/mg/min, 496 +/- 56 nmol O_2_/mg/min, and 19 +/- 2.1 for leak state, ADP-stimulated, and RCR, respectively [10, 43–47, 50–53]. For mitochondria energized with S/Rot, our data using B1 are 101 +/- 9 nmol O_2_/mg/min, 395 +/- 38 nmol O_2_/mg/min, and 4.0 +/- 0.3 for leak state respiration, ADP-stimulated respiration, and the RCR, respectively. This is comparable to results obtained in other studies [47–49]. With PC/M, our data using B1 are 34 +/- 5 nmol O_2_/mg/min, 331 +/- 23 nmol O_2_/mg/min, and 10 +/- 1.2, for leak state respiration, ADP-stimulated respiration, and the RCR, respectively. These data are supported by previous results [49]. Major differences observed between reports are attributed to species differences, tissue source, and quality of the mitochondrial preparation. So in general, our results using a KCl-based buffer are consistent with the previously reported data.

**Table 2:** Mitochondrial respiration data for various common substrates^a^.

Substrate^b^	Leak state	ADP-stimulated	RCR	Reference
P/M (SD)	49 +/- 11	487 +/- 63	9.9	[12]
P (SEM)	11 +/- 1.5	217 +/- 3.54	19.7	[42]
P (SEM)	N/A	N/A	11.6 +/- 0.3	[14]
P (SD)	35 +/- 15.5	342 +/- 48.6	12 +/- 4.8	[43]
G/M (SEM)	40 +/- 3.4	256 +/- 20	6.4	[10]
G/M (SEM)	N/A	279 +/- 3	N/A	[44]
G/M (SEM)	14.5 +/- 1.6	178 +/- 2.7	12.3	[45]
G/M (SEM)	21	131 +/- 10	6.6	[46]
G/M (SEM)	53 +/- 8	217 +/- 15	4.5 +/- 0.2	[47]
G (SD)	38 +/- 14.1	401 +/- 54.5	11 +/- 2.8	[43]
S (SEM)	117 +/- 9	235 +/- 10	2.0 +/- 0.2	[47]
S/Rot (SEM)	N/A	N/A	2.4 +/- 0.2	[48]
S/Rot (SEM)	66 +/- 7	327 +/- 20	4.95	[49]
PC/M (SEM)	36 +/- 5	188 +/- 4	5.3	[49]

^a^Respiratory rate units are in nmol O_2_/mg/min.

^b^standard deviation, SD; standard error of the mean, SEM

We also found that buffers B1 and B2 had significantly different levels of calcium contamination. This lead to differences in the respiratory rates and RCR values when the substrate combination trials were done in the absence of EGTA. As expected, the substrate combinations (G/M, αKG/M, αKG/G) involved with the calcium-sensitive metabolic pathways [11, 12, 54, 55] had higher leak state respiration rates when the calcium levels were elevated (see Fig 5). While pyruvate oxidation is also calcium-sensitive [56, 57], we saw no significant increase in either leak state or ADP-stimulated respiration. We attribute this lack of effect to the high pyruvate concentrations used in the P/M combination respiratory trials. The levels used are high enough to activate the pyruvate dehydrogenase complex in the absence of calcium [58]. The depressed ADP-stimulated respiratory rates for PC/M and αKG/M seen with buffer B1 is surprising. It is possible that the contamination levels of calcium for these substrate combinations is too high and leads to mitochondrial dysfunction as seen by others [59]. Why only these two substrates combinations showed a significant depression in ADP-stimulated rates is unknown and is currently under investigation.

We stress that our calcium results do not contradict other calcium studies that show positive calcium effects on ADP-stimulated respiration [11, 12]. In these studies, the experimental design explicitly sought to characterize the effect of calcium on mitochondrial respiration and energetics. As such, the calcium concentrations were much lower and tightly controlled. In our calcium-dependent studies, we did not seek to control the extra-mitochondrial calcium concentration; therefore, the calcium contaminate was taken up and distributed among the mitochondria. If we assume, on average, the mitochondria lowered the buffer calcium to approximately 0.5–1 μM [60–62], the total calcium load would be 47–52 nmol/mg in buffer B1 and 0–5 nmol/mg in buffer B2. The calcium loads in buffer B1 far exceed those necessary to stimulate the calcium-sensitive matrix dehydrogenases and approach the levels that trigger mitochondrial permeability transition in the absence of adenine nucleotides and/or cyclosporin A [63]. The calcium loads in buffer B2 are at, or slightly above, levels necessary for calcium stimulation [64]. As our experiments were not designed to fully ascertain the effect of calcium on mitochondrial metabolism, we did not purposefully deplete our mitochondria of calcium. This is often done to detect calcium-dependent stimulation [11, 64–66]. Moreover, the effect of calcium on ADP-stimulated respiration is not monotonic, and there are several reports showing a decrease in respiration as the mitochondrial calcium load increases [59, 64, 67]. Therefore, our results are consistent with these findings. The mechanism of inhibition is unknown and under current investigation.

The source of calcium is most likely the reagents used to make up the buffers. This is of no concern when the calcium is controlled with excess EGTA as was done when comparing buffers B1, B2, B3, and B4. However, when studying the impact of specific calcium loads on mitochondrial function, these levels need to be quantified. The lower levels found in B2 are due to lactobionate's calcium chelating properties [21] and can be beneficial when designing mitochondrial calcium-loading experiments. Unfortunately, the formation calcium-lactobionate precipitation can prevent loading of moderate to high calcium concentrations and must be considered when testing mitochondrial function at different calcium loads. A possible strategy is to load the mitochondria with several small calcium chloride boluses instead of a single larger calcium chloride bolus. We did not measure calcium contamination levels in EGTA free gluconate-based buffers (B3 and B4), but we expect them to be higher than the levels found in the lactobionate buffer (B2) considering the calcium association constant is lower for calcium-gluconate relative to calcium-lactobionate [21]. That said, calcium-gluconate precipitation will be an even worse problem for calcium loading experiments, as its solubility is much lower than the solubility of calcium-lactobionate [21].

Regarding the higher ADP-stimulated and FCCP-stimulated respiration rates, we identified several factors present in the tested respiratory buffers that could explain the differences observed between buffers. These are ionic strength, phosphate availability, antioxidant properties of taurine, the presence of sucrose, stability inducing properties of lactobionate, or simply the lower chloride concentrations. While ionic strength differences are known to affect mitochondrial respiration rates [31, 68], their magnitude of effect is not sufficient to explain the findings reported here. In buffers B1 and B3, the total phosphate concentration was 5 mM, but it was 10 mM in buffers B2 and B4. Higher ADP stimulated rates can be attributed to the higher availability of phosphate for phosphorylation reactions, however; we saw no major differences between the ADP-stimulated rate for buffers B2, B3, and B4. These findings are further supported by studies showing that in the presence of saturating ADP, the effect of phosphate is near saturation at 5 mM [10, 69]. It could be possible that the antioxidant and other properties of taurine could lead to significant changes in the ADP-stimulated rates. Nevertheless, our tests using buffers B1 and B2 with and without taurine show that this is not the case. Sucrose can also be ruled out as the determining factor, as buffer B2 and B3 yielded near identical results. And the fact that the gluconate and lactobionate based buffers led to near identical results, suggests that the beneficial effect of lactobionate is non-exclusive. The remaining factor is simply the lower chloride concentrations. Chloride is known to inhibit the creatine kinase reaction [28], but our respiratory trials did not include the need for this enzyme. In addition, chloride has been shown to inhibit the adenine nucleotide translocase by preventing adenine nucleotide binding to the translocase [26, 27]. These findings fit nicely with our results. Moreover, our data reveal additional targets of chloride-dependent inhibition on mitochondrial function. Specifically, the FCCP-stimulated respiration rates were still lower for the G/M, αKG/M, αKG/G, PC/M, and S/Rot substrate combinations. We are unaware of studies that have investigated chloride inhibition of other solute carriers such as the dicarboxylate carrier, alpha-ketoglutarate/malate exchanger, or beta-oxidation related processes, but our results seem to suggest these mitochondrial transporters and enzymes are also subject to chloride inhibition. Interestingly, FCCP-stimulated respiration on P/M was not significantly different between buffers. Therefore, the monocarboxylate transporter responsible for pyruvate transport did not appear to be as detrimentally affected by high chloride concentrations as other transporters.

The choice between lactobionate and gluconate as the anionic replacement for chloride in respiratory buffers is left up to the experimenter. While our respiratory trials show no significant difference between the two chloride substitutes, there may be certain advantages of using one over the other. For example, lactobionate may possess several beneficial properties for mitochondria [18, 70]. Lactobionate also has calcium-chelating properties, which helped decrease calcium contamination levels in buffer B2 without EGTA [20, 22]. And prior research has found that lactobionate acts to decrease cellular injury in preserved tissue for organ transplantation, but the mechanism has yet to be demonstrated [18, 70]. In addition to lower chloride concentrations, the lactobionate- and gluconate-based buffers could also improve mitochondrial function by favoring a more condensed, energy-transducing, mitochondrial state associated with ADP stimulated respiration [71, 72]. However, one drawback with lactobionate is the formation of calcium-lactobionate precipitation at moderate to high calcium concentrations. With the gluconate, calcium precipitation occurs at even lower calcium concentrations. This precipitation hinders testing the mitochondrial permeability transition phenomenon using buffers containing these anions; however, a simple work-around is to load mitochondrial with smaller doses of calcium over a longer timeframe versus a single addition of a large calcium dose. Nevertheless, respirations buffers containing high levels of chloride should be avoided when investigating respiratory function and ATP production by isolated mitochondria.

## Conclusions

In conclusion, we show that traditional KCl-based respiratory buffers contain inhibitory levels of chloride that can lower both ADP-stimulated and FCCP-stimulated respiration. A broad range of substrates consisting of all the major substrates oxidized by mitochondria in various combinations that targeted both NADH and UQH_2_ driven respiration were tested. We found that chloride not only inhibited adenine nucleotide translocase activity, but it also appeared to inhibit substrate transport across the inner mitochondrial membrane. Specifically, succinate, alpha-ketoglutarate, glutamate, and palmitoylcarnitine transport and oxidation were affected but pyruvate transport was less affected. Therefore, if the goal is to maximize the rate of oxidative phosphorylation to test mitochondrial function, the buffers containing low levels of chloride are highly recommended.

## Supporting information

S1 Fig Representative respiration dynamics for each tested substrate combination in the presence of low levels of free calcium. Buffers B1 and B2 were modified by removing EGTA from the constituents which resulted in small amounts of free calcium contamination. The calcium contamination levels were 5.7 +/- 0.51 μM (n = 5) of free calcium in B1 and 0.99 +/- 0.27 μM (n = 4) in B2. About 2.5 μM EGTA was added with the addition of mitochondria to the chamber as carryover from the 1 mM EGTA in the isolation buffer. When the leak state respiration rate stabilized, 500 μM ADP was added to initiate oxidative phosphorylation. The elevated respiration rates after ADP phosphorylation is due to variable contamination of ATPases from the isolation process. Respiration dynamics for P/M, G/M, αKG/M, αKG/G, PC/M, P/M/S, and S/Rot supported respiration are shown. The respiration dynamics are aligned to the time of ADP addition at five minutes. The insets give the corresponding oxygen concentration data.
(TIFF)

S2 Fig ADP-stimulated and FCCP-stimulated respiration rates relative to B1. The data in Fig 2 for the ADP-stimulated (A) and FCCP-stimulated (B) respiration rates for buffers B2, B3, and B4 are replotted as a percent difference compared to B1. This comparison further supports the conclusion that chloride inhibits the adenine nucleotide translocase (and/or the phosphate carrier and/or the F_1_F_O_ ATP synthase) in addition to possible substrate transporters for succinate, alpha-ketoglutarate, glutamate, and palmitoylcarnitine. Error bars are propagated standard deviations
(TIF)
