# Supplementary figures and images for: The effect of respiration buffer composition on mitochondrial metabolism and function
Source: PLoS One. 2017 Nov 1;12(11):e0187523. doi: 10.1371/journal.pone.0187523 (PMC5665555; doi:10.1371/journal.pone.0187523)

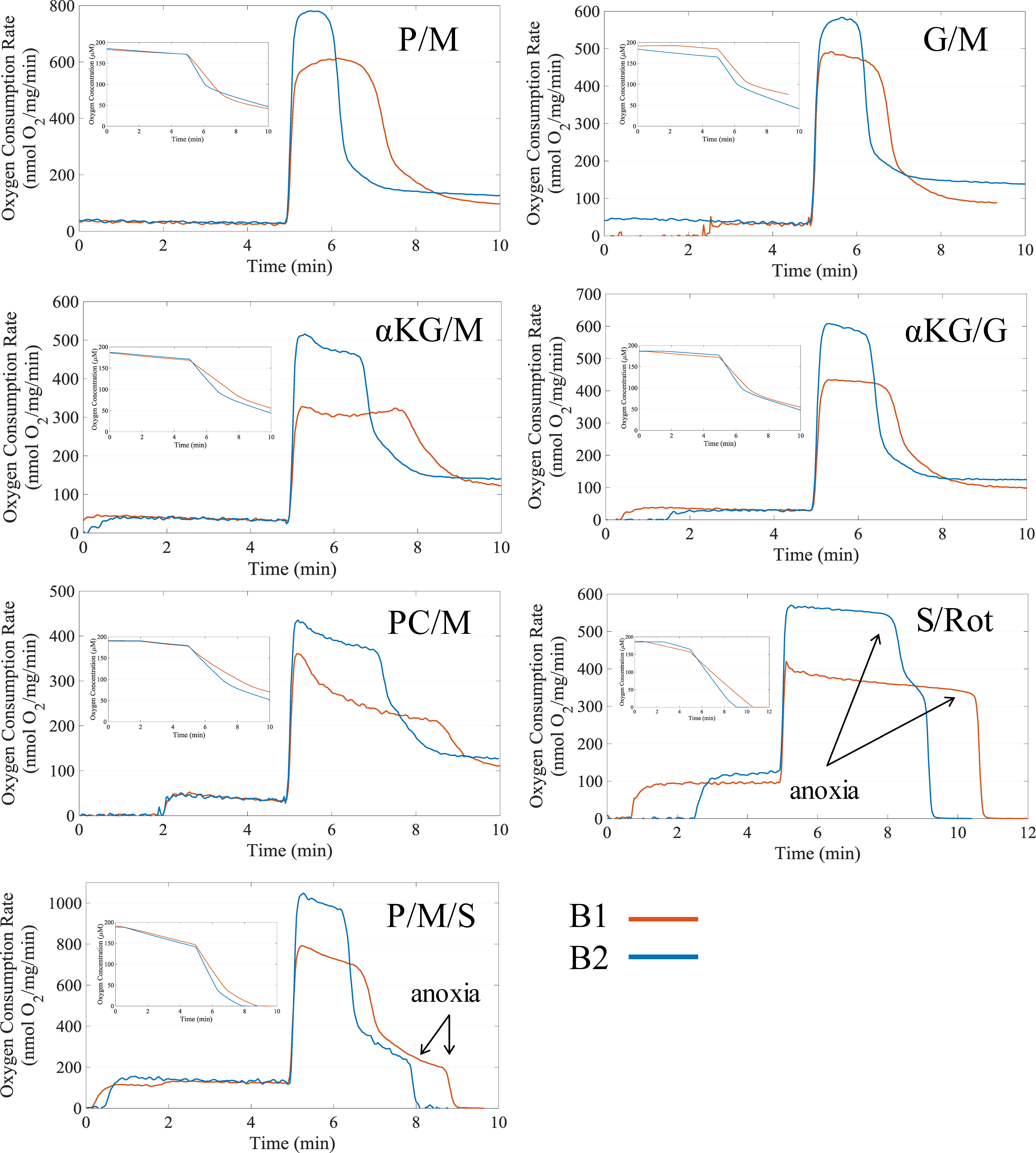

Supplement: S1 Fig — Buffers B1 and B2 were modified by removing EGTA from the constituents which resulted in small amounts of free calcium contamination. The calcium contamination levels were 5.7 +/- 0.51 μM (n = 5) of free calcium in B1 and 0.99 +/- 0.27 μM (n = 4) in B2. About 2.5 μM EGTA was added with the addition of mitochondria to the chamber as carryover from the 1 mM EGTA in the isolation buffer. When the leak state respiration rate stabilized, 500 μM ADP was added to initiate oxidative phosphorylation. The elevated respiration rates after ADP phosphorylation is due to variable contamination of ATPases from the isolation process. Respiration dynamics for P/M, G/M, αKG/M, αKG/G, PC/M, P/M/S, and S/Rot supported respiration are shown. The respiration dynamics are aligned to the time of ADP addition at five minutes. The insets give the corresponding oxygen concentration data. (TIFF) [file pone.0187523.s001.tiff]

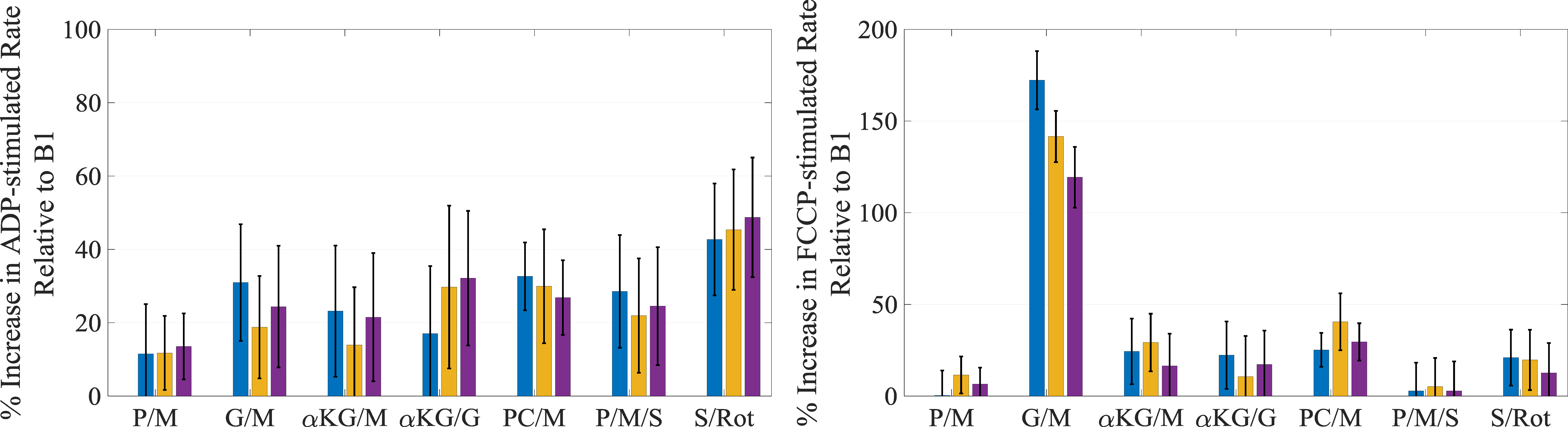

Supplement: S2 Fig — The data in Fig 2 for the ADP-stimulated (A) and FCCP-stimulated (B) respiration rates for buffers B2, B3, and B4 are replotted as a percent difference compared to B1. This comparison further supports the conclusion that chloride inhibits the adenine nucleotide translocase (and/or the phosphate carrier and/or the F1FO ATP synthase) in addition to possible substrate transporters for succinate, alpha-ketoglutarate, glutamate, and palmitoylcarnitine. Error bars are propagated standard deviations (TIF) [file pone.0187523.s002.tif]
